# Supplementary material for: Modeling glioblastoma heterogeneity as a dynamic network of cell states
Source: Mol Syst Biol. 2021 Sep 16;17(9):e10105. doi: 10.15252/msb.202010105 (PMC8444284; doi:10.15252/msb.202010105)
Supplement: Supplementary file 5 — Source Data for Figure 3 [file MSB-17-e10105-s001.zip › Figure3A_sourcedata/GSEA_3065/nosigdb_state2.GseaPreranked.1622718419215/gsea_report_for_na_pos_1622718419215.html]

Report for na\_pos 1622718419215 [GSEA]

| GS  follow link to MSigDB | GS DETAILS | SIZE | ES | NES | NOM p-val | FDR q-val | FWER p-val | RANK AT MAX | LEADING EDGE || 1 | LARSSON\_STATE\_4 | Details ... | 101 | 1.00 | 3.34 | 0.000 | 0.000 | 0.000 | 100 | tags=100%, list=1%, signal=100% |
| 2 | DOROSZKO\_U3180MG\_CLUSTER\_3 | Details ... | 483 | 0.86 | 3.30 | 0.000 | 0.000 | 0.000 | 977 | tags=78%, list=10%, signal=82% |
| 3 | DOROSZKO\_U3013MG\_CLUSTER\_2 | Details ... | 210 | 0.91 | 3.30 | 0.000 | 0.000 | 0.000 | 644 | tags=85%, list=7%, signal=89% |
| 4 | RICHARDS\_ZHONG\_NATURE\_2018\_NPCS\_UPREG | Details ... | 126 | 0.91 | 3.09 | 0.000 | 0.000 | 0.000 | 514 | tags=79%, list=5%, signal=82% |
| 5 | FELDMAN\_2020\_G1 | Details ... | 149 | 0.87 | 3.03 | 0.000 | 0.000 | 0.000 | 514 | tags=86%, list=5%, signal=89% |
| 6 | NB\_KI\_CLUSTER5 | Details ... | 86 | 0.93 | 3.01 | 0.000 | 0.000 | 0.000 | 519 | tags=91%, list=5%, signal=95% |
| 7 | FELDMAN\_2020\_S-G2 | Details ... | 330 | 0.80 | 3.00 | 0.000 | 0.000 | 0.000 | 602 | tags=68%, list=6%, signal=70% |
| 8 | GAROFANO\_IAVARONE\_PPR\_SUBTYPE | Details ... | 449 | 0.78 | 2.97 | 0.000 | 0.000 | 0.000 | 1021 | tags=52%, list=11%, signal=56% |
| 9 | SEURAT\_CELLCYCLE | Details ... | 93 | 0.90 | 2.96 | 0.000 | 0.000 | 0.000 | 831 | tags=91%, list=9%, signal=99% |
| 10 | FELDMAN\_2020\_S | Details ... | 151 | 0.84 | 2.93 | 0.000 | 0.000 | 0.000 | 630 | tags=72%, list=7%, signal=76% |
| 11 | RICHARDS\_NOWAKOWSKI\_SCIENCE\_2017\_RG\_EARLY\_UPREG | Details ... | 441 | 0.72 | 2.75 | 0.000 | 0.000 | 0.000 | 1166 | tags=48%, list=12%, signal=52% |
| 12 | FELDMAN\_2020\_G2-M | Details ... | 289 | 0.73 | 2.71 | 0.000 | 0.000 | 0.000 | 831 | tags=63%, list=9%, signal=67% |
| 13 | RICHARDS\_NOWAKOWSKI\_SCIENCE\_2017\_MGE\_RG2\_UPREG | Details ... | 445 | 0.69 | 2.66 | 0.000 | 0.000 | 0.000 | 1223 | tags=52%, list=13%, signal=57% |
| 14 | SUVA\_G2M\_PMID\_31327527 | Details ... | 45 | 0.88 | 2.62 | 0.000 | 0.000 | 0.000 | 644 | tags=82%, list=7%, signal=88% |
| 15 | SUVA\_G1S\_PMID\_31327527 | Details ... | 27 | 0.96 | 2.59 | 0.000 | 0.000 | 0.000 | 342 | tags=96%, list=4%, signal=100% |
| 16 | RICHARDS\_NEFTEL\_CELL\_2019\_G2\_M | Details ... | 45 | 0.88 | 2.58 | 0.000 | 0.000 | 0.000 | 644 | tags=82%, list=7%, signal=88% |
| 17 | DALMO\_BARCODENUMBER\_UNPUB | Details ... | 192 | 0.72 | 2.58 | 0.000 | 0.000 | 0.000 | 641 | tags=54%, list=7%, signal=56% |
| 18 | RICHARDS\_NEFTEL\_CELL\_2019\_G1\_S | Details ... | 27 | 0.96 | 2.57 | 0.000 | 0.000 | 0.000 | 342 | tags=96%, list=4%, signal=100% |
| 19 | GAROFANO\_IAVARONE\_LIST\_OF\_NEURAL\_PROGENITOR\_MARKER\_GENES | Details ... | 87 | 0.77 | 2.53 | 0.000 | 0.000 | 0.000 | 633 | tags=53%, list=7%, signal=56% |
| 20 | DOROSZKO\_U3180MG\_CLUSTER\_4 | Details ... | 170 | 0.69 | 2.44 | 0.000 | 0.000 | 0.000 | 1548 | tags=59%, list=16%, signal=69% |
| 21 | IVY\_CT |  | 148 | 0.68 | 2.39 | 0.000 | 0.000 | 0.000 | 375 | tags=39%, list=4%, signal=39% |
| 22 | DOROSZKO\_U3054MG\_CLUSTER\_6 |  | 119 | 0.70 | 2.36 | 0.000 | 0.000 | 0.000 | 2014 | tags=66%, list=21%, signal=83% |
| 23 | NB\_KI\_CLUSTER3 |  | 163 | 0.64 | 2.27 | 0.000 | 0.000 | 0.000 | 2482 | tags=69%, list=26%, signal=92% |
| 24 | FELDMAN\_2020\_NEURAL G0 |  | 371 | 0.59 | 2.22 | 0.000 | 0.000 | 0.000 | 725 | tags=39%, list=8%, signal=40% |
| 25 | OLSEN\_ADRENERGIC |  | 65 | 0.70 | 2.20 | 0.000 | 0.000 | 0.000 | 938 | tags=52%, list=10%, signal=58% |
| 26 | NB\_KI\_CLUSTER1 |  | 32 | 0.74 | 2.08 | 0.000 | 0.000 | 0.000 | 1967 | tags=84%, list=21%, signal=106% |
| 27 | RICHARDS\_NOWAKOWSKI\_SCIENCE\_2017\_MGE\_IPC3\_UPREG |  | 119 | 0.61 | 2.08 | 0.000 | 0.000 | 0.000 | 1199 | tags=39%, list=13%, signal=44% |
| 28 | FELDMAN\_2020\_LATEG1 |  | 88 | 0.63 | 2.07 | 0.000 | 0.000 | 0.000 | 238 | tags=32%, list=3%, signal=32% |
| 29 | FELDMAN\_2020\_M-EARLYG1 |  | 109 | 0.61 | 2.04 | 0.000 | 0.000 | 0.000 | 731 | tags=47%, list=8%, signal=50% |
| 30 | VANGRONINGEN\_ADRN |  | 237 | 0.51 | 1.86 | 0.000 | 0.000 | 0.010 | 734 | tags=21%, list=8%, signal=22% |
| 31 | PARADA\_T2\_UP\_26461091 |  | 67 | 0.58 | 1.83 | 0.000 | 0.000 | 0.026 | 708 | tags=31%, list=7%, signal=34% |
| 32 | PARADA\_T1\_UP\_26461091 |  | 64 | 0.57 | 1.78 | 0.001 | 0.001 | 0.062 | 708 | tags=42%, list=7%, signal=45% |
| 33 | SINGH\_SOX2\_UPREGULATED\_TRANSCRIPTS\_PMID\_28122245 |  | 427 | 0.41 | 1.55 | 0.000 | 0.015 | 0.685 | 708 | tags=14%, list=7%, signal=15% |
| 34 | WANG\_PRONEURAL\_PMID\_28697342 |  | 15 | 0.62 | 1.49 | 0.038 | 0.026 | 0.883 | 1384 | tags=47%, list=15%, signal=55% |
| 35 | NB\_KI\_CLUSTER4 |  | 93 | 0.44 | 1.46 | 0.017 | 0.033 | 0.940 | 907 | tags=27%, list=10%, signal=29% |
| 36 | ALMSTEDT\_RISK |  | 30 | 0.47 | 1.28 | 0.147 | 0.128 | 1.000 | 228 | tags=27%, list=2%, signal=27% |
| 37 | RICHARDS\_NOWAKOWSKI\_SCIENCE\_2017\_IPC\_NEN3\_UPREG |  | 231 | 0.33 | 1.22 | 0.093 | 0.181 | 1.000 | 1590 | tags=29%, list=17%, signal=34% |
| 38 | RICHARDS\_VERHAAK\_CANCERCELL\_2010\_PRONEURAL |  | 116 | 0.34 | 1.15 | 0.200 | 0.280 | 1.000 | 647 | tags=15%, list=7%, signal=16% |
| 39 | RICHARDS\_NOWAKOWSKI\_SCIENCE\_2017\_IN\_CTX\_CGE1\_UPREG |  | 296 | 0.30 | 1.14 | 0.168 | 0.299 | 1.000 | 2119 | tags=29%, list=22%, signal=36% |
| 40 | ALMSTEDT\_11Q\_MSIGDB |  | 312 | 0.29 | 1.09 | 0.277 | 0.388 | 1.000 | 2017 | tags=27%, list=21%, signal=33% |
| 41 | ALMSTEDT\_17Q\_MSIGDB |  | 363 | 0.29 | 1.08 | 0.287 | 0.395 | 1.000 | 1560 | tags=18%, list=17%, signal=21% |
| 42 | ROSEN\_KRONA\_TI\_UNPUBL |  | 54 | 0.35 | 1.06 | 0.380 | 0.430 | 1.000 | 1129 | tags=17%, list=12%, signal=19% |
| 43 | RICHARDS\_NOWAKOWSKI\_SCIENCE\_2017\_IN\_CTX\_MGE1\_UPREG |  | 279 | 0.27 | 1.01 | 0.450 | 0.529 | 1.000 | 1478 | tags=23%, list=16%, signal=27% |
| 44 | WENG\_STEMNESS\_PMID\_30982771 |  | 36 | 0.34 | 0.95 | 0.555 | 0.644 | 1.000 | 408 | tags=14%, list=4%, signal=14% |
| 45 | GANDAL\_SCHIZOPHRENIA\_DN\_PMID29439242 |  | 17 | 0.39 | 0.93 | 0.573 | 0.681 | 1.000 | 1912 | tags=35%, list=20%, signal=44% |
| 46 | RICHARDS\_NOWAKOWSKI\_SCIENCE\_2017\_NIN4\_UPREG |  | 131 | 0.24 | 0.83 | 0.816 | 0.861 | 1.000 | 1451 | tags=21%, list=15%, signal=24% |
| 47 | GANDAL\_DEPRESSION\_DN\_PMID29439242 |  | 34 | 0.26 | 0.74 | 0.858 | 0.975 | 1.000 | 2173 | tags=21%, list=23%, signal=27% |
| 48 | NB\_KI\_CLUSTER9 |  | 25 | 0.27 | 0.72 | 0.844 | 0.977 | 1.000 | 3593 | tags=48%, list=38%, signal=77% |
| 49 | RICHARDS\_VERHAAK\_CANCERCELL\_2010\_NEURAL |  | 63 | 0.22 | 0.68 | 0.951 | 0.981 | 1.000 | 1845 | tags=19%, list=20%, signal=24% |
| 50 | NB\_KI\_CLUSTER7 |  | 304 | 0.14 | 0.53 | 1.000 | 0.997 | 1.000 | 2213 | tags=15%, list=23%, signal=19% |
Table: Gene sets enriched in phenotype **na **[plain text format]****
